# Feasibility of a best–worst scaling exercise to set priorities for autism research

**DOI:** 10.1111/hex.13508

**Published:** 2022-06-08

**Authors:** Scott A. Davis, Kirsten Howard, Alan R. Ellis, Daniel E. Jonas, Timothy S. Carey, Joseph P. Morrissey, Kathleen C. Thomas

**Affiliations:** ^1^ Division of Pharmaceutical Outcomes and Policy, Eshelman School of Pharmacy University of North Carolina Asheville North Carolina USA; ^2^ Faculty of Medicine and Health, School of Public Health, Faculty of Medicine and Health University of Sydney Sydney Australia; ^3^ Department of Sociology North Carolina State University Raleigh North Carolina USA; ^4^ Department of Internal Medicine Ohio State University Medical Center Columbus Ohio USA; ^5^ Department of Epidemiology, Gillings School of Global Public Health University of North Carolina Chapel Hill North Carolina USA; ^6^ Cecil G. Sheps Center for Health Services Research Chapel Hill North Carolina USA; ^7^ Department of Health Policy and Management, Gillings School of Global Public Health University of North Carolina Chapel Hill North Carolina USA

**Keywords:** autism, best–worst scaling, direct prioritization, discrete‐choice experiment, stakeholder engagement, stakeholder priorities

## Abstract

**Background:**

The preferences of autism stakeholders regarding the top priorities for future autism research are largely unknown.

**Objective:**

This study had two objectives: First, to examine what autism stakeholders think new research investments should be and the attributes of investment that they consider important, and second, to explore the feasibility, acceptability and outcomes of two prioritization exercises among autism stakeholders regarding their priorities for future research in autism.

**Design:**

This was  a prospective stakeholder‐engaged iterative study consisting of best–worst scaling (BWS) and direct prioritization exercise.

**Setting and Participants:**

A national snowball sample of 219 stakeholders was included: adults with autism, caregivers, service providers and researchers.

**Main Outcome Measures:**

The main outcomes measures were attributes that participants value in future research investments, and priority research investments for future research.

**Results:**

Two hundred and nineteen participants completed the exercises, of whom 11% were adults with autism, 58% were parents/family members, 37% were service providers and 21% were researchers. Among stakeholders, the BWS exercises were easier to understand than the direct prioritization, less frequently skipped and yielded more consistent results. The proportion of children with autism affected by the research was the most important attribute for all types of stakeholders. The top three priorities among future research investments were (1) evidence on which child, family and intervention characteristics lead to the best/worst outcomes; (2) evidence on how changes in one area of a child's life are related to changes in other areas; and (3) evidence on dietary interventions. Priorities were similar for all stakeholder types.

**Conclusions:**

The values and priorities examined here provide a road map for investigators and funders to pursue autism research that matters to stakeholders.

**Patient or Public Contribution:**

Stakeholders completed a BWS and direct prioritization exercise to inform us about their priorities for future autism research.

## 1 INTRODUCTION

Autism spectrum disorders affect about 1 in 54 Americans, and their prevalence continues to rise over time.^1^ Systematic reviews continue to suggest a shortage of evidence to definitively inform the best treatment practices for children with autism.^2^, ^3^, ^4^, ^5^, ^6^ Stakeholder input could help with setting future research priorities, but few researchers have systematically gathered input from individuals with autism and their caregivers to inform future funding priorities.

Systematic reviews have noted that relatively few high‐quality randomized‐controlled trials have examined autism treatments; therefore, it is not possible to say with certainty that any treatment is effective or ineffective, only that more research needs to be done.^4^, ^6^ There is weak evidence for behavioural treatment of autism and somewhat stronger evidence that medications such as aripiprazole can help with problem behaviours,^2^, ^3^, ^4^ suggesting that future effectiveness research should focus on strengthening the evidence on these treatments. However, medications can have harmful side effects, and stakeholders may prefer treatments that do not involve medication.^4^, ^5^, ^7^ Also, stakeholders sometimes have considerable interest in treatments with which they have had a positive experience, but for which only limited or anecdotal evidence currently exists.^8^ Knowing more about stakeholder preferences might help resolve some of the uncertainty about what kind of additional evidence should be generated from research to benefit children with autism. Therefore, our first aim was to examine what autism stakeholders think new research investments should be and the attributes of investment that they consider important.

Prioritization exercises have shown great promise in eliciting public values and preferences to inform healthcare decisions made by public officials and healthcare institutions.^9^, ^10^, ^11^, ^12^ Choice‐based preference elicitation methods such as discrete‐choice experiments (DCEs) and best–worst scaling (BWS) exercises are being used with increasing frequency to gain information about stakeholder preferences and priorities for research.^9^, ^10^, ^11^, ^12^ There is limited research using choice‐based methods such as DCEs or BWS with autism stakeholders.^13^ One DCE by Dosreis et al.^13^ included caregivers of children with various developmental disabilities, including autism, but no published DCEs have involved individuals with autism as respondents.^13^ Therefore, it is unclear whether DCEs are a meaningful approach to capture preferences for adults with autism and other autism stakeholders. Therefore, our second aim was methodological: to explore the feasibility, acceptability and outcomes of two prioritization exercises with autism stakeholders, one based on a BWS exercise and the other a direct prioritization activity, regarding future research in autism. In pursuing the second aim, we also hoped to enhance the evidence base concerning the use of these methods with consumers and the public in prioritizing future research investments in healthcare generally.

## 2 METHODS

### 2.1 Study design

The design was a prospective stakeholder‐engaged iterative study. A survey instrument, developed with stakeholder input in an iterative process from formulation to finalization of the document (*N* = 17), captured priorities for future research. Three separate pairs of focus groups comprised of adults with autism (*n* = 6), parents (*n* = 5) and professionals (*n* = 6) were conducted to develop the survey. Recruitment was accomplished through clinical contacts, and participants were diverse with respect to age and race. We met with each stakeholder group separately so that participants would feel free to express opinions that might not be shared by other stakeholder types, such as the need for pharmaceutical treatment. We met with each group twice. In the first meeting, we discussed values that underlie treatment choice. In the second meeting, we reviewed the list of values and discussed future research investments. The product of these focus groups included lists of values and investments that stakeholders agreed were complete and meaningful. The survey consisted of two parts: A BWS experiment to examine how much autism stakeholders valued each of nine attributes derived from stakeholder value statements of future research investments and a direct prioritization exercise. The direct prioritization exercise had two subsections: First, stakeholders ranked each of the nine attributes from the BWS experiment, and second, stakeholders ranked 17 future research directions from highest to lowest priority.^14^

### 2.2 Survey participants

The survey was completed by a range of autism stakeholders: adults with autism, parents, providers and researchers. The survey was conducted in person at regular meeting groups of stakeholders in North Carolina, Georgia, California and Colorado and via the web among a national snowball sample of autism stakeholders. In‐person survey implementation was conducted by two people: a social scientist (Dr. Thomas) and a research assistant. We invited participation in the web‐based survey through national advocacy groups, such as Autism Speaks, The Autism Society of America and Mental Health America; internet blogs by adults with autism; professional groups such as the Association of University Centers on Disabilities; state Developmental Disabilities Councils; the Study to Explore Early Development in autism; and numerous individual and tweeted invitations to complete and share the survey. Participants in the in‐person setting received $100 in thanks for their efforts, while participants in the web survey did not receive payment. Data collection occurred between May and December 2014.

### 2.3 Measures

In the BWS exercise, 9 two‐level attributes described what people value in future research investments, based on qualitative work conducted before the survey. Sample attributes include ‘the treatment BUILDS ON STRENGTHS of the child with autism’ versus ‘the treatment FOCUSES ON REDUCING PROBLEMS with autism rather than building on strengths’ and ‘the treatment addresses MULTIPLE ASPECTS OF AUTISM in an integrated manner’ versus ‘the treatment focuses on a SINGLE ASPECT OF AUTISM’ (Appendix SA). A fractional factorial design (balanced incomplete block design) was used to keep the number of attributes presented in each question to five, to make the process easier for participants.^10^ Stakeholders completed 18 questions, each of which asked the participant to choose their most favourite and least favourite attribute among a group of five hypothetical aspects (i.e., attributes) of a certain research scenario; the five attributes presented varied across the questions.

In the direct prioritization exercise, participants were shown the nine attributes from the BWS exercise and 17 future research investments to prioritize.^14^ Examples of research investments from the BWS exercise include ‘Evidence on which child, family, and intervention characteristics lead to the best (and worst) outcomes’, ‘Evidence on dietary interventions’ and ‘Evidence on behavioural interventions’ (Appendix SA). Stakeholders completed two questions, one of which asked them to directly rate the nine attributes from the BWS exercise, and one of which asked them to rate the 17 potential areas for future research. Each participant had a total of six stars (priority votes) available to distribute among the nine attributes in the first question, and 12 stars available to distribute among the 17 areas for future research investment in the second question. A maximum of four stars could be assigned to any single priority.

In the in‐person settings, we described the goals of the study and our interest in learning which type of prioritization exercise worked best. At the close of the survey exercise, we discussed reactions. Discussions were audio‐recorded and transcribed. We used open coding to assess question‐asking about our methodology and reactions.^15^, ^16^

### 2.4 Analysis

To assess the feasibility and acceptability of the exercises for respondents, we recorded questions during implementation and feedback from discussion. We also calculated missingness in each type of prioritization exercise and rates of completion. For the direct prioritization exercise, rankings of attributes and future research investments were calculated, both overall and by subgroup, from the mean number of stars allocated to each attribute or investment.

To model participants' choices from the BWS exercise, we estimated a multinomial logit (MNL) model in NLOGIT 5.0. Each attribute level was coded as 1 when it was chosen as most favourite (‘best’), −1 when it was chosen as least favourite (‘worst’) and 0 when the attribute level was not chosen as either most or the least favourite. Therefore, in the MNL model, each person contributes observations for ‘best’ (1 or 0), ‘worst’ (−1 or 0) for each attribute in each scenario, yielding up to a total of 180 observations per respondent—18 scenarios of five attributes * 2 choices (best or worst).^10^ Coefficients for attribute level were estimated from the combined data set of the ‘best’ and the ‘worst’ choices, assuming that a best choice mirrors a worst choice and that there are no positive or negative framing effects.^17^ The core multinomial logit model was Choice = *β*_0_ + *β*_1_bw_indic + *β*_2..9_attributes + *β*_10..26_attribute‐levels. Choice indicates whether the given attribute level was chosen. Bw_indic indicates whether the outcome is a choice of ‘best’ (rather than ‘worst’). *β*_2..9_ are the attribute coefficients (eight coefficients are estimated – nine attributes minus the attribute assigned as the reference attribute), and *β*_10..26_ are the attribute‐level coefficients for attribute‐level combinations (17 coefficients estimated – 9 attributes of two levels each (18), minus one attribute level assigned as reference attribute level). In results, reference categories of attributes and attribute levels are presented as 0. We explored differences in preferences between stakeholder types (adults with autism, parents and professionals) by running the same multinomial logit model within each subgroup. Parameter estimates describe participants' preferences for one attribute or attribute level compared with a reference attribute and attribute level. A statistically significant coefficient indicates the importance of an attribute level in determining overall utility and in influencing preferences. Attribute importance (interpreted as the overall impact of an attribute on utility) is calculated from the range of *β* across levels (*β*_max_ − *β*_min_ for each attribute). The priority, or utility, of a given future research investment is the sum of the predicted log odds of choosing its attributes and attribute levels^10^, ^18^ and is calculated by multiplying the *β* for each attribute level by the actual attribute level present in that research investment, and then summing all the products.

We compared rankings of future research investments, as well as individual attributes, between the direct prioritization and BWS using Spearman correlations. The rankings for the BWS were based on attribute importance and priority/utility calculations described above.

## 3 RESULTS

Stakeholders in North Carolina, Georgia, California and Colorado completed the survey in person (*n* = 101), and 118 completed the web survey, for a total of 219 participants. The demographics of the participants are shown in Table 1. The mean age was 43 years (SD = 11), with 11% of the stakeholders being adults with autism, 58% being parents or family members, 37% being service providers and 21% being researchers. The majority were female (79%) and non‐Hispanic White (77%). Participants resided in 23 states across all four regions of the United States.

**Table 1:** Demographics of participants (*N* = 219)

Characteristics	*n*	Percent or mean (SD)
Stakeholder role^a^		
Adult with autism	25	11%
Parent or other family member	127	58%
Service provider	82	37%
Researcher	47	21%
Individuals with two or more roles		
Parent and provider	36	16%
Parent and researcher	12	5%
Provider and researcher	18	8%
Any two or more	62	28%
Autism age group of interest^b^		
0–10 years	149	68%
11–20 years	147	67%
20+ years	137	63%
All	90	41%
Autism functioning level of interest^a^		
Low functioning	130	59%
High functioning	184	84%
Both	112	51%
Female	172	79%
Age	219	43 (11)
Race and ethnicity		
White not Hispanic	169	77%
Black not Hispanic	10	5%
Other not Hispanic	27	12%
Hispanic	13	6%
Education^c^		
No college degree	42	19%
College degree	68	31%
Graduate degree	100	46%
Region^c^		
Northeast	18	8%
Midwest	11	5%
South	109	50%
West	76	35%

^a^ Participants could choose more than one category.

^b^ The autism age groups appear exactly as listed on the survey.

^c^ There were missing values for education (*n* = 9) and region (*n* = 5).

Postsurvey discussions after in‐person implementation gave people a chance to convey what they felt was important; to consider differences of opinion, experience, past need and current need; and to affirm the value of each other's opinions. These discussions typically lasted a full hour after completion of the paper survey. The group format encouraged people to complete the survey and rewarded them with an opportunity for expression that the web survey lacked. Stakeholders reported that they found the discussion of research attributes engaging. Participants expressed enthusiasm for the research topic and eagerness to discuss what they valued and what they felt were important knowledge gaps and important future research investments. Participants stated that the BWS exercises were easier to understand than the direct prioritization. They asked questions to understand directions more frequently in direct prioritization. In‐person notes and recordings did not allow for distinction of stakeholder type in these discussions.

Compared with the direct prioritization questions, the BWS scenarios also were less frequently skipped (10% vs. 26% missing items on average) and yielded more consistent results between stakeholder types. However, stakeholders expressed dislike for the forced‐choice aspect of BWS. Participants were more likely to complete the survey in the in‐person setting than on the web. Missing responses and completion rates did not vary by stakeholder type.

Table 2 shows the multinomial logit model results for the full sample. The attributes with the highest utility were whether the research addresses multiple aspects of autism in an integrated manner and whether the proportion of children with autism affected by the knowledge gained was high, while the attributes with the lowest utility were that the out‐of‐pocket cost of the treatment was high and the proportion of children with autism affected by the knowledge gained was low. There was a statistically significant difference between every pair of levels.

**Table 2:** Multinomial logit results of BWS for attributes and their levels

Attribute (attribute level)	Coefficient	*p*
*The proportion of children with autism affected by the research*	0.2611	.0579
The proportion of children with autism affected by the knowledge gained is LOW, <50%	0.86389	<.00001
The proportion of children with autism affected by the knowledge gained is HIGH, ≥50%	6.0979	<.00001
*The cost of the knowledge to be gained*	−0.00498	.9653
The cost of the research to gain the desired knowledge is LOW, <$5 MILLION	3.83683	<.00001
The cost of the research to gain the desired knowledge is HIGH, ≥$5 MILLION	1.58321	<.00001
*The age of the children who would benefit from the treatment*	0.26375	.0050
The children who would benefit from the treatment are YOUNG, <11	3.04534	<.00001
The children who would benefit from the treatment are OLDER, ≥11	4.04334	<.00001
*The focus of the treatment on the child and support environment*	0.13896	.2268
The treatment focuses on the CHILD with autism ALONE	2.55912	<.00001
The treatment focuses on the CHILD with autism AND his or her SUPPORT ENVIRONMENT such as family, school and health service providers	5.65729	<.00001
*The cost of the treatment developed that families pay out of pocket*	−0.22809	.0745
The cost per week of the treatment developed that families pay out of pocket is HIGH, ≥$500 PER WEEK	0	
The cost per week of the treatment developed that families pay out of pocket is LOW, <$500 PER WEEK	4.60737	<.00001
*Addresses autism and disorders that often occur with autism*	0	
The treatment addresses BOTH AUTISM AND OTHER DISORDERS that often occur with autism	5.31251	<.00001
The treatment addresses AUTISM ONLY, NOT OTHER DISORDERS that often occur with autism	2.3311	<.00001
*Addresses multiple aspects of autism in an integrated manner*	0.05327	.6994
The treatment addresses MULTIPLE ASPECTS OF AUTISM in an integrated manner	6.15433	<.00001
The treatment focuses on a SINGLE ASPECT OF AUTISM	1.75883	<.00001
*Builds on the strengths of the child with autism*	0.33914	.0004
The treatment BUILDS ON THE STRENGTHS of the child with autism	5.28117	<.00001
The treatment FOCUSES ON REDUCING PROBLEMS of autism rather than building on strengths	3.01987	<.00001
*Develops life skills*	0.40849	<.00001
The treatment DEVELOPS LIFE SKILLS	4.66066	<.00001
The treatment FOCUSES ON SYMPTOMS rather than developing life skills	3.44546	<.00001
*Likelihood ratio statistic*	*15,087*	*<.00001*

Abbreviation: BWS, best–worst scaling.

### 3.1 Attribute importance

**Figure 1:**
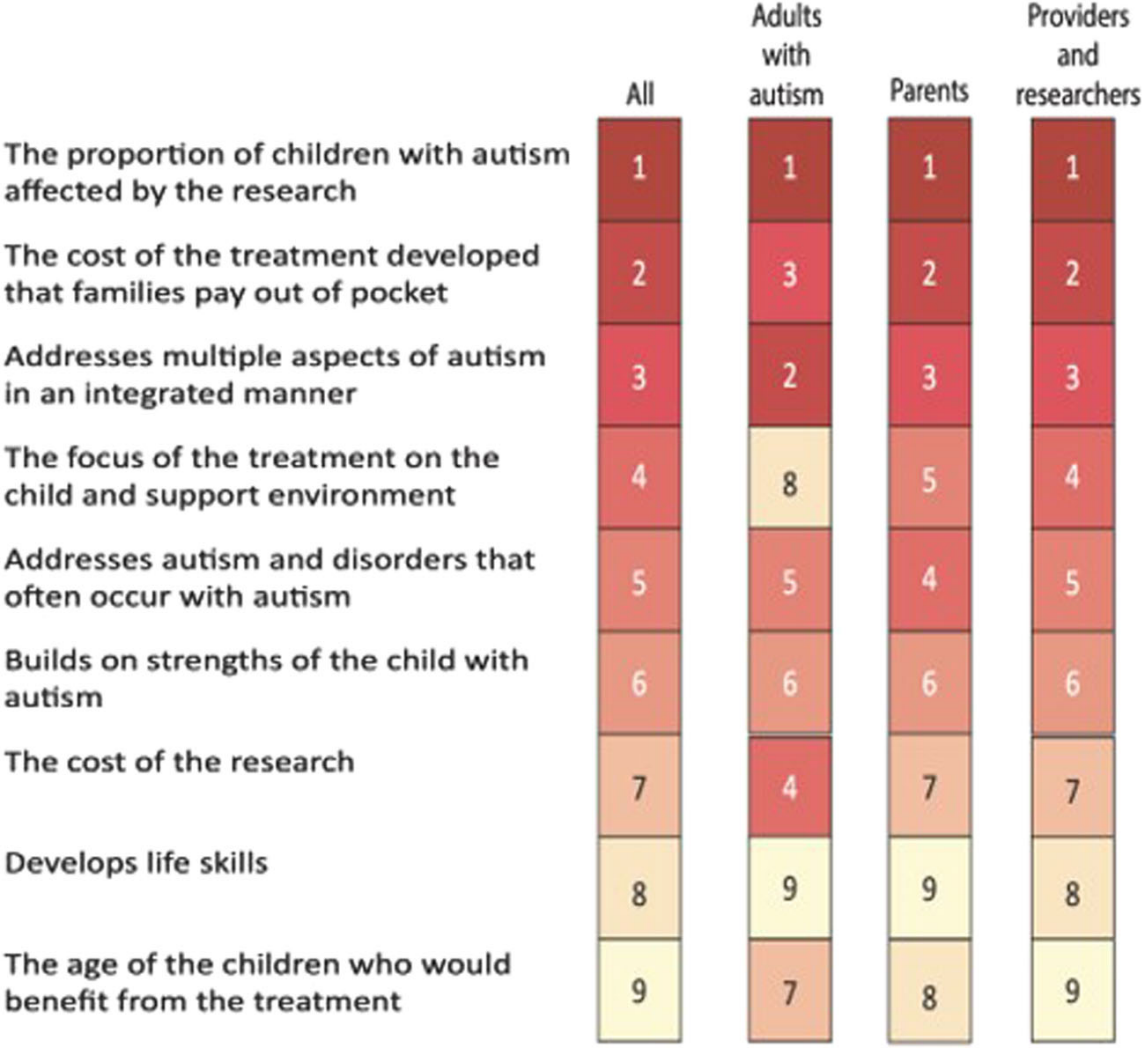
Priority of attributes of autism research from best–worst scaling. *Numbers and shading reflect the rank of the given population*

Figure 1 shows the rankings of the nine attributes of research priorities in autism estimated from the BWS exercise overall and by stakeholder type. All types of stakeholders agreed that the top priority was the proportion of children with autism affected by the research. Overall, stakeholders selected the proportion of children with autism affected by the research, family out‐of‐pocket costs for the treatment, whether or not the treatment addresses multiple aspects of autism in an integrated manner and the focus of the treatment on the child and support environment as the most valued attributes. The main differences were that adults with autism rated the cost of the research fourth, compared to seventh for the other stakeholder types, and adults with autism rated the focus of the treatment on the child and support environment eighth, compared to fifth and fourth for parents and providers/researchers, respectively.

The direct prioritization method yielded overall attribute rankings (not shown) that were consistent with the rankings from the full‐sample BWS model (*r* = .70, *p* = .03). In the direct prioritization exercise, as in the BWS model, stakeholders selected the proportion of children with autism affected by the research as the most valued attribute. Among adults with autism, the direct prioritization identified treatment that develops life skills and treatment that builds on strengths as neither highest nor lowest valued, differing somewhat from the BWS, but otherwise consistent with the BWS.

### 3.2 Future research investment ranking

**Figure 2:**
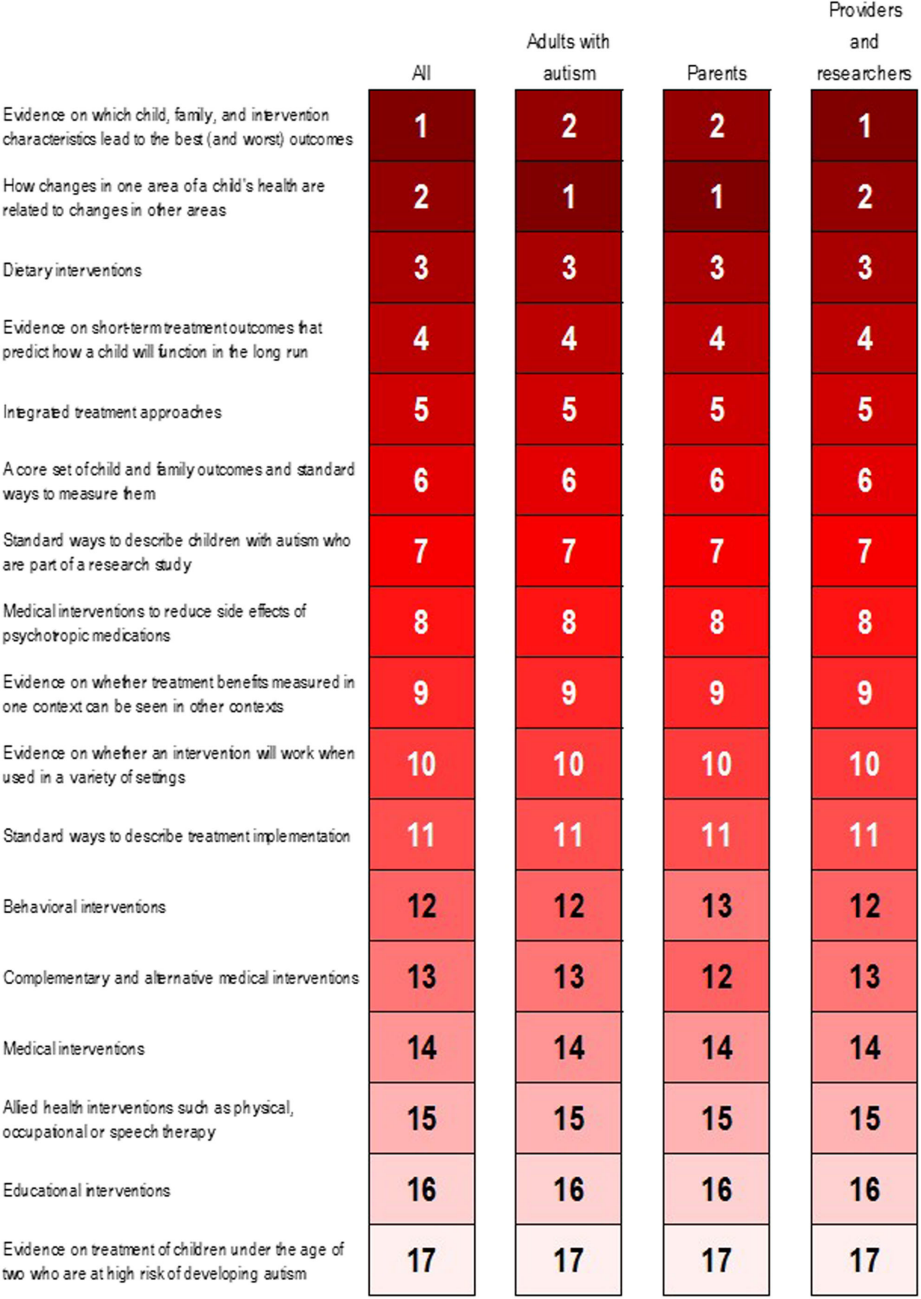
Future research Investment priorities from best–worst scaling. *Numbers and shading reflect the rank of the given population*

Using the attribute and level betas from the multinomial logit model to estimate the overall value of a future research investment, Figure 2 shows the rankings of the 17 future research Investments for each stakeholder type from the BWS for the full population and by subpopulation. The top three future research Investments were (1) evidence on which child, family and intervention characteristics lead to the best (and worst) outcomes; (2) evidence on how changes in one area of a child's life are related to changes in other areas; and (3) evidence on dietary interventions.

The direct prioritization exercise identified the same top stakeholder priority overall for future autism research, but yielded results that generally differed from those of the BWS exercise for some of the other priorities (*r* = .24, *p* = .54). The second and third priorities under the direct prioritization approach were (2) evidence on integrated treatment approaches, in which multiple treatments are combined in a comprehensive manner, and (3) evidence on behavioural interventions.

## 4 DISCUSSION

First, research that would benefit a high percentage of children with autism was the most valued attribute of future autism research by all types of stakeholders. Both methods that were used produced similar priorities. The data did not suggest major differences between the priorities of researchers and those of other stakeholder types. Out‐of‐pocket cost of the treatment and the ability to address multiple aspects of autism in an integrated manner were attributes of significant concern for all stakeholders as well. Better evidence could help to show which treatments are worth the cost in light of the benefits gained by children with autism and by society. Systematic reviews have rated the quality of evidence for early intensive behavioural intervention as low because most of the evidence came from nonrandomized studies.^4^, ^6^ Our study suggests that higher‐quality evidence on behavioural treatments would be welcomed by autism stakeholders of all kinds, given that effective behavioural treatments would affect a large percentage of children with autism and would potentially address multiple aspects of autism in an integrated manner.

There was the greatest consensus on the top research investment priorities: evidence on which child/family/intervention characteristics leads to the best and worst outcomes, how changes in different areas of a child's life are related and dietary interventions. It is worth noting that some priorities are very expensive, both in terms of requiring large long‐term trials to establish effectiveness, as well as likely costs of paying professionals to deliver treatment. Future research should give stakeholders more specific information on the cost of research to see whether their preferences remain the same when they are fully informed about research costs and trade‐offs with other research activities. Considering stakeholder priorities free of cost considerations and taking them into account are critical for collaborative policy‐making. Without attention to stakeholder priorities and trade‐offs, adults with autism have frequently felt that they did not have a voice in their interactions with the healthcare system.^7^, ^19^ Additionally, engaging stakeholders in setting priorities can help funding agencies to stay grounded in the lives of those affected by research.^20^ For example, a study by Frazier et al.^21^ noted that a minority of autism stakeholders wanted to avoid most or all types of autism research, especially studies of genetic markers or prenatal screening. Although we did not observe this, our methodology might not have been ideally suited to identifying areas where stakeholders opposed specific research investments.^21^, ^22^ Basic science studies, such as those on genetic causes of autism, were beyond the scope of our treatment‐focused research choices.^21^, ^22^, ^23^

Second, this study demonstrated the feasibility of using a BWS exercise to determine the preferences of autism stakeholders regarding priority areas for future research and attributes that future research would ideally have. We found that, compared with direct prioritization, BWS was easier for participants to understand and generated important discussions of the values underlying stakeholders' research priorities, with less missing data. This finding is consistent with evidence that BWS was an easy and effective method for discerning caregiver priorities regarding their children's mental health.^9^, ^24^ BWS provides advantages over simple Likert‐scale survey methods because it requires respondents to choose one priority over others, while keeping cognitive burden relatively low.^16^, ^25^ The similarity of our BWS and direct prioritization results supports using the less burdensome BWS method in future research to understand the preferences of individuals with autism and their caregivers. For future research, these methods will need to be refined so that rankings can be given context from the stakeholders' personal perspectives, ensuring that stakeholders have been able to fully express what matters most to them. For example, a larger sample with more detailed information about neighbourhood context might reveal different preferences between stakeholders in resource‐rich environments (who might be eager for new and varied treatments) and stakeholders in resource‐poor environments (who may have had negative experiences from limited treatment options). A larger sample with more details on household context might allow examination of variation in preferences based on the extent of medical mistrust and experience of structural racism. While our initial work has shown that the DCE approach is promising, it will require more research to maximize the information gained from autism stakeholders.

There were a few limitations of the study. Only 11% of the study population were people with autism; however, parents and other family members made up 58% of the study population, and preferences of adults with autism did not differ greatly from those of other participants. We recruited participants mainly through advocacy groups, professional groups and social media. Future research should attempt to determine whether stakeholders who are not highly engaged in these organizations or in social media may have different preferences. Research using samples derived from registries or through electronic health record searches might be one way to address this issue. The study was performed in 2014, so some stakeholder preferences might differ today. However, there have been no new systematic reviews on autism treatment. When a new systematic review is warranted, it will likely identify somewhat different knowledge gaps, so this exercise should be periodically repeated as new systematic review evidence is accumulated since this can aid funders in prioritizing their research funding announcements. The study also has notable strengths; to our knowledge, this is the first BWS to include multiple types of autism stakeholders, including adults with autism, to prioritize future research investments.

Both the highest‐ranked attribute priority, concerning the proportion of children with autism affected, and the highest‐ranked research investment priority, concerning evidence on which child, family and intervention characteristics lead to the best and worst outcomes, relate to heterogeneity of treatment effect, which is a key part of precision medicine. Autism has a very broad range of presentations, and no single treatment seems to work for everyone.^2^, ^3^, ^4^, ^5^, ^6^ These findings emphasize that evidence is needed on how best to tailor the available treatments for individuals.

## 5 CONCLUSION

Although conceptualizing future research is challenging, all stakeholders valued having a voice in the discussion of future research investment and findings were similar across stakeholder types (adults with autism, parents and professionals). BWS worked better than direct prioritization for these autism stakeholders. Findings provided a ranking of research attributes and investment priorities that can help provide a road map for researchers and funders to engage stakeholders in identifying specific research questions and pursue research that matters to stakeholders.

## AUTHOR CONTRIBUTIONS

Drs. Howard, Ellis, Jonas, Carey, Morrissey and Thomas participated in the study design. Drs. Howard and Thomas participated in the acquisition of data. Drs. Davis, Howard and Thomas participated in the drafting of the final manuscript. All authors participated in the critical review of the manuscript. All authors have given final approval to the manuscript.

## CONFLICTS OF INTEREST

The authors declare no conflicts of interest.

## Supporting information

Supporting information.

## Data Availability

Research data are not shared.
