# Supplementary material for: Feasibility of a best–worst scaling exercise to set priorities for autism research
Source: Health Expect. 2022 Jun 8;25(4):1643–51. doi: 10.1111/hex.13508 (PMC9327819; doi:10.1111/hex.13508)
Supplement: Supplementary file 1 — Supporting information. [file HEX-25--s001.docx]

**Appendix: Web Survey**

**Exploring values for future research in autism**

**The goal of this survey**

This survey is about future research on the treatment of childhood autism. We want to learn what features you value in that research.

**Survey Structure**

Part I asks some brief questions about you.

Part II presents a set of questions about what you value in autism research.

Part III asks you to rank research features and possible directions.

This survey should take about 30 minutes to complete.

**Contact Information**

This research is funded by the Patient-Centered Outcomes Research Institute and conducted by the University of North Carolina at Chapel Hill.

If you have any questions, please contact Kathleen Thomas at 919-966-3387 or [Kathleen_thomas@unc.edu](mailto:Kathleen_thomas@unc.edu).

Part I

The first few questions are about you and your role as an autism stakeholder.

**1. What autism stakeholder roles do you fill?** *Check all that apply.*

___ adult with autism

___ parent or other family member

___ service provider

___ researcher

___ don’t know or prefer not to answer

**2. In your role as an autism stakeholder, in what age groups are you interested?** *Check all that apply.*

___ 0-10 years

___ 11-20 years

___ 20+ years

___ don’t know or prefer not to answer

**3. In your role as an autism stakeholder, in what levels of functioning are you interested?** *Check all that apply.*

___ low functioning autism

___ high functioning autism

___ don’t know or prefer not to answer

**4. In which state do you live?**

_______________________

___ don’t know or prefer not to answer

**5. What is your gender?**

___ female

___ male

___ don’t know or prefer not to answer

**6. What is your age?**

___ years

___ don’t know or prefer not to answer

**7. What is your race?**

___White

___Black or African American

___American Indian or Alaska Native

___Asian

___Native Hawaiian or other Pacific Islander

___other

___ don’t know or prefer not to answer

**8. Are you of Hispanic or Latino origin?**

___ Yes

___ No

___ don’t know or prefer not to answer

**9. What is your highest level of education?**

___ Never attended

___ Elementary

___ Some high school

___ High school graduate

___ Some college or technical school

___ College graduate

___ Graduate degree

___ don’t know or prefer not to answer

Part II

The following questions are about your opinions regarding the direction of future research in the treatment of childhood autism and what features you value in that research.

We will present you with a set of questions that describe several features of future research in the treatment of childhood autism. We’ll ask you to indicate your least favorite thing and most favorite thing.

The questions will look very similar to each other, but each is different. Please check your least and most favorite feature for each question. Below is an example question about what people value in different places to live.

In this example, a living place was described by four features: the person’s least favorite thing about this place was that it is far from work. The person’s most favorite thing about this place was that it is close to family or friends.

**Example: What is your least favorite and most favorite thing about where you live?**

| **Least favorite thing** | **Example question** | **Most favorite thing** |
| --- | --- | --- |
| 🗹 | It is far from work | ☐ |
| ☐ | It is close to family or friends | 🗹 |
| ☐ | It is affordable | ☐ |
| ☐ | It is far from shopping | ☐ |

**In the next series of questions, we will ask you to indicate your least favorite thing and most favorite thing about different kinds of research about autism treatment.**

Future research in the treatment of childhood autism will be described by 9 features:

Two features describe the knowledge to be gained:

1. The proportion of children with autism affected by the research

2. The cost of the research

The remaining features describe the treatment that would be developed:

3. The age of the children who would benefit from the treatment

4. The focus of the treatment

5. The out-of-pocket cost of the treatment

6. The ability of the treatment to address disorders that often go along with autism

7. The ability of the treatment to address multiple aspects of autism

8. The strategy of the treatment

9. The ability of the treatment to improve life skills

**1. What is your least favorite and most favorite thing about this research?**

| **Least favorite thing** |  | **Most favorite thing** |
| --- | --- | --- |
| ☐ | FEW children with autism would be affected by the knowledge gained | ☐ |
| ☐ | The treatment focuses ONLY ON THE CHILD WITH AUTISM | ☐ |
| ☐ | The out of pocket cost of the treatment would be LOW | ☐ |
| ☐ | The treatment addresses MULTIPLE ASPECTS OF AUTISM | ☐ |
| ☐ | The treatment FOCUSES ON IMPROVING SYMPTOMS rather than life skills | ☐ |

**2. What is your least favorite and most favorite thing about this research?**

| **Least favorite thing** |  | **Most favorite thing** |
| --- | --- | --- |
| ☐ | The cost of the research would be LOW | ☐ |
| ☐ | The out of pocket cost of the treatment would be LOW | ☐ |
| ☐ | The treatment addresses BOTH AUTISM AND OTHER DISORDERS that often go along with autism | ☐ |
| ☐ | The treatment focuses on a SINGLE ASPECT OF AUTISM | ☐ |
| ☐ | The treatment BUILDS ON STRENGTHS of the child with autism rather than focusing on problems | ☐ |

**3. What is your least favorite and most favorite thing about this research?**

| **Least favorite thing** |  | **Most favorite thing** |
| --- | --- | --- |
| ☐ | The treatment being studied would benefit CHILDREN UNDER AGE 11 | ☐ |
| ☐ | The treatment focuses ON THE CHILD WITH AUTISM AND SUPPORT PEOPLE such as family, school and health service providers | ☐ |
| ☐ | The treatment addresses BOTH AUTISM AND OTHER DISORDERS that often go along with autism | ☐ |
| ☐ | The treatment BUILDS ON STRENGTHS of the child with autism rather than focusing on problems | ☐ |
| ☐ | The treatment FOCUSES ON IMPROVING LIFE SKILLS rather than symptoms | ☐ |

**4. What is your least favorite and most favorite thing about this research?**

| **Least favorite thing** |  | **Most favorite thing** |
| --- | --- | --- |
| ☐ | FEW children with autism would be affected by the knowledge gained | ☐ |
| ☐ | The treatment being studied would benefit CHILDREN AGES 11 AND UP | ☐ |
| ☐ | The treatment focuses ONLY ON THE CHILD WITH AUTISM | ☐ |
| ☐ | The treatment focuses on a SINGLE ASPECT OF AUTISM | ☐ |
| ☐ | The treatment BUILDS ON STRENGTHS of the child with autism rather than focusing on problems | ☐ |

**5. What is your least favorite and most favorite thing about this research?**

| **Least favorite thing** |  | **Most favorite thing** |
| --- | --- | --- |
| ☐ | FEW children with autism would be affected by the knowledge gained | ☐ |
| ☐ | The cost of the research would be HIGH | ☐ |
| ☐ | The out of pocket cost of the treatment would be HIGH | ☐ |
| ☐ | The treatment BUILDS ON STRENGTHS of the child with autism rather than focusing on problems | ☐ |
| ☐ | The treatment FOCUSES ON IMPROVING LIFE SKILLS rather than symptoms | ☐ |

**6. What is your least favorite and most favorite thing about this research?**

| **Least favorite thing** |  | **Most favorite thing** |
| --- | --- | --- |
| ☐ | The cost of the research would be HIGH | ☐ |
| ☐ | The treatment being studied would benefit CHILDREN UNDER AGE 11 | ☐ |
| ☐ | The treatment addresses BOTH AUTISM AND OTHER DISORDERS that often go along with autism | ☐ |
| ☐ | The treatment focuses on a SINGLE ASPECT OF AUTISM | ☐ |
| ☐ | The treatment FOCUSES ON IMPROVING SYMPTOMS rather than life skills | ☐ |

**7. What is your least favorite and most favorite thing about this research?**

| **Least favorite thing** |  | **Most favorite thing** |
| --- | --- | --- |
| ☐ | MANY children with autism would be affected by the knowledge gained | ☐ |
| ☐ | The cost of the research would be HIGH | ☐ |
| ☐ | The treatment focuses ON THE CHILD WITH AUTISM AND SUPPORT PEOPLE such as family, school and health service providers | ☐ |
| ☐ | The treatment addresses AUTISM ONLY, NOT OTHER DISORDERS that often go along with autism | ☐ |
| ☐ | The treatment focuses on a SINGLE ASPECT OF AUTISM | ☐ |

**8. What is your least favorite and most favorite thing about this research?**

| **Least favorite thing** |  | **Most favorite thing** |
| --- | --- | --- |
| ☐ | The cost of the research would be HIGH | ☐ |
| ☐ | The treatment being studied would benefit CHILDREN UNDER AGE 11 | ☐ |
| ☐ | The treatment focuses ON THE CHILD WITH AUTISM AND SUPPORT PEOPLE such as family, school and health service providers | ☐ |
| ☐ | The out of pocket cost of the treatment would be LOW | ☐ |
| ☐ | The treatment FOCUSES ON REDUCING PROBLEMS of autism rather than building on strengths | ☐ |

**9. What is your least favorite and most favorite thing about this research?**

| **Least favorite thing** |  | **Most favorite thing** |
| --- | --- | --- |
| ☐ | MANY children with autism would be affected by the knowledge gained | ☐ |
| ☐ | The treatment being studied would benefit CHILDREN AGES 11 AND UP | ☐ |
| ☐ | The out of pocket cost of the treatment would be LOW | ☐ |
| ☐ | The treatment addresses AUTISM ONLY, NOT OTHER DISORDERS that often go along with autism | ☐ |
| ☐ | The treatment FOCUSES ON IMPROVING LIFE SKILLS rather than symptoms | ☐ |

**10. What is your least favorite and most favorite thing about this research?**

| **Least favorite thing** |  | **Most favorite thing** |
| --- | --- | --- |
| ☐ | FEW children with autism would be affected by the knowledge gained | ☐ |
| ☐ | The cost of the research would be LOW | ☐ |
| ☐ | The treatment being studied would benefit CHILDREN UNDER AGE 11 | ☐ |
| ☐ | The treatment addresses AUTISM ONLY, NOT OTHER DISORDERS that often go along with autism | ☐ |
| ☐ | The treatment FOCUSES ON REDUCING PROBLEMS of autism rather than building on strengths | ☐ |

**11. What is your least favorite and most favorite thing about this research?**

| **Least favorite thing** |  | **Most favorite thing** |
| --- | --- | --- |
| ☐ | FEW children with autism would be affected by the knowledge gained | ☐ |
| ☐ | The cost of the research would be LOW | ☐ |
| ☐ | The treatment being studied would benefit CHILDREN AGES 11 AND UP | ☐ |
| ☐ | The treatment focuses ON THE CHILD WITH AUTISM AND SUPPORT PEOPLE such as family, school and health service providers | ☐ |
| ☐ | The treatment FOCUSES ON IMPROVING SYMPTOMS rather than life skills | ☐ |

**12. What is your least favorite and most favorite thing about this research?**

| **Least favorite thing** |  | **Most favorite thing** |
| --- | --- | --- |
| ☐ | MANY children with autism would be affected by the knowledge gained | ☐ |
| ☐ | The cost of the research would be LOW | ☐ |
| ☐ | The treatment being studied would benefit CHILDREN UNDER AGE 11 | ☐ |
| ☐ | The out of pocket cost of the treatment would be HIGH | ☐ |
| ☐ | The treatment addresses MULTIPLE ASPECTS OF AUTISM | ☐ |

**13. What is your least favorite and most favorite thing about this research?**

| **Least favorite thing** |  | **Most favorite thing** |
| --- | --- | --- |
| ☐ | The cost of the research would be LOW | ☐ |
| ☐ | The treatment focuses ONLY ON THE CHILD WITH AUTISM | ☐ |
| ☐ | The out of pocket cost of the treatment would be HIGH | ☐ |
| ☐ | The treatment addresses AUTISM ONLY, NOT OTHER DISORDERS that often go along with autism | ☐ |
| ☐ | The treatment FOCUSES ON IMPROVING LIFE SKILLS rather than symptoms | ☐ |

**14. What is your least favorite and most favorite thing about this research?**

| **Least favorite thing** |  | **Most favorite thing** |
| --- | --- | --- |
| ☐ | The treatment being studied would benefit CHILDREN AGES 11 AND UP | ☐ |
| ☐ | The treatment focuses ON THE CHILD WITH AUTISM AND SUPPORT PEOPLE such as family, school and health service providers | ☐ |
| ☐ | The out of pocket cost of the treatment would be HIGH | ☐ |
| ☐ | The treatment addresses BOTH AUTISM AND OTHER DISORDERS that often go along with autism | ☐ |
| ☐ | The treatment addresses MULTIPLE ASPECTS OF AUTISM | ☐ |

**15. What is your least favorite and most favorite thing about this research?**

| **Least favorite thing** |  | **Most favorite thing** |
| --- | --- | --- |
| ☐ | MANY children with autism would be affected by the knowledge gained | ☐ |
| ☐ | The treatment focuses ONLY ON THE CHILD WITH AUTISM | ☐ |
| ☐ | The out of pocket cost of the treatment would be LOW | ☐ |
| ☐ | The treatment addresses BOTH AUTISM AND OTHER DISORDERS that often go along with autism | ☐ |
| ☐ | The treatment FOCUSES ON REDUCING PROBLEMS of autism rather than building on strengths | ☐ |

**16. What is your least favorite and most favorite thing about this research?**

| **Least favorite thing** |  | **Most favorite thing** |
| --- | --- | --- |
| ☐ | The treatment being studied would benefit CHILDREN AGES 11 AND UP | ☐ |
| ☐ | The out of pocket cost of the treatment would be HIGH | ☐ |
| ☐ | The treatment focuses on a SINGLE ASPECT OF AUTISM | ☐ |
| ☐ | The treatment FOCUSES ON REDUCING PROBLEMS of autism rather than building on strengths | ☐ |
| ☐ | The treatment FOCUSES ON IMPROVING SYMPTOMS rather than life skills | ☐ |

**17. What is your least favorite and most favorite thing about this research?**

| **Least favorite thing** |  | **Most favorite thing** |
| --- | --- | --- |
| ☐ | MANY children with autism would be affected by the knowledge gained | ☐ |
| ☐ | The treatment addresses AUTISM ONLY, NOT OTHER DISORDERS that often go along with autism | ☐ |
| ☐ | The treatment addresses MULTIPLE ASPECTS OF AUTISM | ☐ |
| ☐ | The treatment BUILDS ON STRENGTHS of the child with autism rather than focusing on problems | ☐ |
| ☐ | The treatment FOCUSES ON IMPROVING SYMPTOMS rather than life skills | ☐ |

**18. What is your least favorite and most favorite thing about this research?**

| **Least favorite thing** |  | **Most favorite thing** |
| --- | --- | --- |
| ☐ | The cost of the research would be HIGH | ☐ |
| ☐ | The treatment focuses ONLY ON THE CHILD WITH AUTISM | ☐ |
| ☐ | The treatment addresses MULTIPLE ASPECTS OF AUTISM | ☐ |
| ☐ | The treatment FOCUSES ON REDUCING PROBLEMS of autism rather than building on strengths | ☐ |
| ☐ | The treatment FOCUSES ON IMPROVING LIFE SKILLS rather than symptoms | ☐ |

Part III.

In the last two questions, we’d like to ask you directly about the factors you value and directions for future research in autism.

**1. Rank the features of future research in autism by placing stars next to the features that you value. The more stars you add, the higher you rank the feature.**

**You have 6 stars; you can place up to 4 stars per line.** *To add stars, color in the stars in the box to the right of the features.*

| **1. The proportion of children with autism affected by the research** |  |
| --- | --- |
| **2. The cost of the research** |  |
| **3. The age of the children who would benefit from the treatment** |  |
| **4. Whether the treatment focuses only on the child with autism OR on the child with autism and support people such as family, school and health service providers** |  |
| **5. The out of pocket cost of the treatment** |  |
| **6. The ability of the treatment to address disorders that often go along with autism** |  |
| **7. The ability of the treatment to address multiple aspects of autism** |  |
| **8. Whether the treatment builds on strengths of the child with autism rather than focusing on problems OR the treatment focuses on reducing problems of autism rather than building on strengths** |  |
| **9. Whether the treatment focuses on improving life skills rather than symptoms OR the treatment focuses on improving symptoms rather than life skills** |  |

**2. Rank the directions for future research in autism by placing stars next to the research that you value. The more stars you add, the higher you rank the research. You have 12 stars; you can place up to 4 stars per line.** *To add stars, color in the stars in the box to the right of the features.*

| **1. Collect evidence on integrated treatment approaches, in which multiple treatments are combined in a comprehensive manner** |  |
| --- | --- |
| **2. Collect evidence on behavioral interventions** |  |
| **3. Collect evidence on educational interventions** |  |
| **4. Collect evidence on medical interventions** |  |
| **5. Collect evidence on medical interventions to reduce side effects of psychotropic medications** |  |
| **6. Collect evidence on dietary interventions** |  |
| **7. Collect evidence on allied health interventions such as physical, occupational or speech therapy** |  |
| **8. Collect evidence on complementary and alternative medical interventions such as acupuncture or herbal remedies** |  |
| **9. Collect evidence on which child, family, and intervention characteristics lead to the best (and worst) outcomes** |  |
| **10. Collect evidence on how changes in one area of a child’s health (such as anxiety) are related to changes in other areas (such as eating)** |  |
| **11. Collect evidence on short-term treatment outcomes that predict how a child will function in the long run (for example, in terms of life skills)** |  |
| **12. Collect evidence on whether treatment benefits measured in one context (such as home) can be seen in other contexts (such as outside home)** |  |
| **13. Collect evidence on whether an intervention will work when used in a variety of settings (such as school or physician office)** |  |
| **14. Collect evidence on treatment of children under the age of two who are at high risk of developing autism** |  |
| **15. Create a core set of child and family outcomes and standard ways to measure them** |  |
| **16. Create standard ways to describe children with autism who are part of a research study** |  |
| **17. Create standard ways to describe treatment implementation (for example, faithfulness to the treatment manual, quality of care, amount of parent participation)** |  |

**Thank you for giving your time to tell us what you value in future research on autism.**

If you have any questions, please contact Kathleen Thomas at 919-966-3387 or [Kathleen_thomas@unc.edu](mailto:Kathleen_thomas@unc.edu).

If you are interested in participating in additional research on priorities for future research in autism or would like to receive information about our findings, please leave your email below. We will never publish or share your email address.

Email:___________________________________ [optional]
